# Do neighbourhood characteristics matter in understanding school children’s active lifestyles? A cross-region multi-city comparison of Glasgow, Edinburgh and Hong Kong

**DOI:** 10.1080/14733285.2020.1828826

**Published:** 2020-10-21

**Authors:** Jonathan R. Olsen, Kevin Y.K. Leung, Natalie Nicholls, Becky P.Y. Loo

**Affiliations:** a MRC/CSO Social and Public Health Sciences Unit, University of Glasgow, Glasgow, UK; b Department of Geography, University of Hong Kong, Hong Kong, Hong Kong

**Keywords:** After-school activities, everyday life, children, inequality, international

## Abstract

Many studies have explored the influence of individual and neighbourhood factors on active school travel (AST), this novel study is the first to examine how AST and formal extracurricular activities are associated with children’s active lifestyles. The aims of this study were to (a) create an active lifestyle variable (ALIFE) measured in terms of total weekly minutes of AST and extracurricular activities, and (b) explore how ALIFE is associated with different attributes at the individual, household and neighbourhood levels, and how these relationships differ for children aged 10 and 11 years old across the three cities: Glasgow, Edinburgh and Hong Kong. We found environmental factors to be important indicators of lower AST, for example greater parking facility density. The most substantial contribution to children’s overall ALIFE was household income, those from the lowest household group having almost 2 h less ALIFE per-week than those from the highest income.

## Introduction

Over the past decade, there has been increasing attention in the scientific literature directed towards exploring children’s active lifestyles and travel characteristics. The major focus has been on active school travel (AST) (non-motorised transport mode, for example: walking, cycling or human-powered scootering), in Western (Chillón et al. 2009; Mcdonald 2007; Van Goeverden and De Boer 2013; Pojani and Boussauw 2014) and non-Western (Irawan and Sumi 2011; Mori, Armada, and Willcox 2012; De Kadt et al. 2014; Zhang, Yao, and Liu 2017) contexts. Despite the well-documented physiological and psychosocial benefits of AST (Van Sluijs et al. 2009; Lubans et al. 2011; Ramanathan et al. 2014; Leung and Loo 2017), there has been a general decline of AST rates around the world (Prevention 2005; Garrard 2009; Shaw et al. 2015). The second major focus has been on children’s physical activity (PA) as part of their total daily of weekly activities; for example, including both active travelling and participation in sport/play (i.e. PA comprising both discretionary and non-discretionary purposes) (Alexander et al. 2005; Van Sluijs et al. 2009; Lubans et al. 2011). After all, higher levels of PA achieved through AST *and* extra-curricular activities have many benefits for children’s health and fitness (Armstrong and Van Mechelen 2008). However, a notable decline occurs around the time children end primary school (Reilly 2016), which is a major cause for concern among parents, educators and policymakers alike.

An important explanatory factor for AST is the proliferation of neighbourhood environments that prioritise car use over walking, cycling and public transport (Rothman et al. 2018). This causes a vicious cycle between increased traffic flow and increased perceptions of traffic danger, which may impact children’s active travel. Some contributing factors to decreased AST include: (1) increases in car ownership, (2) driving children to school perceived as being more convenient, and (3) school catchment areas (i.e. the geographic area from which children are eligible to attend a local school) increasing, which, in turn, means the average distance children are living from their school has increased (Fyhri et al. 2011). Meanwhile, compact, highly interconnected and walkable neighbourhoods have been shown to encourage rates of both AST and PA in young people (Molina-García et al. 2017; Leung et al. 2019). These neighbourhoods often have better access to sport and leisure facilities, such as parks and playgrounds (Giles-Corti et al. 2009).

Many other factors have been shown to influence AST levels in children. Key factors include sex, where girls have commonly been identified to be less active in travelling to school and partake in shorter durations of sport activities than boys (Mello and Worrell 2010), as well as showing greater reductions in physical activity during adolescence than boys (Dumith et al. 2011). As an individual level, Santos et al. (2009) identified a positive association between lower BMI with participation in guided sports activities. Household-level factors are also shown to be important in predicting AST, for example children are likely to make over half of their school trips with a family member (Mcdonald 2008). Household car ownership has been consistently associated with less active travel to destinations for children (Waygood, Sun, and Letarte 2015; Zhang, Yao, and Liu 2017), and as car ownership is lower for lower income households, this can have an indirect positive impact on health by increasing active travel. Children from middle-income households have been shown to participate in more sport activities out of school than their counterparts of lower socio-economic status (Holloway and Pimlott-Wilson 2014; Karsten 2015). This calls for further work to examine whether the relationship will hold for an active lifestyle concept.

There are other important considerations for AST, as both geographical location and culture may hold differences for AST in children within different contexts globally. For example, the neighbourhood-built environment has been found to have significant importance for AST in children from studies in North America (Mitra and Buliung 2015) and Australia (Timperio et al. 2006). Continental differences between the physical environment and AST were found between North American, Australian and European children (D’haese et al. 2015).

The research described above highlights the importance for further exploration of the built environment and AST between different countries, where consistencies between study design, methods and data collection allow. We have also highlighted that there is little understanding of the influence of children’s formal extracurricular activities on levels of AST, for example after school tutoring, music or sports lessons, as well as cultural differences in these. Much of the literature has examined either AST (e.g.(Lubans et al. 2011; Rothman et al. 2018)) or extracurricular activities individually (e.g. (Covay and Carbonaro 2010; Mello and Worrell 2010; De Meester et al. 2017)). AST *and* extracurricular activities may be associated either negatively or positively on both outcomes through constraints on children’s time, tiredness or through a more active lifestyle. One of the few studies combining AST and extracurricular activities found that taking part in extracurricular activities decreased active travel for Canadian students due to the incurrence of time cost through total activity participation (Wong et al. 2011b).

This study is innovative in being the first that compares and contrasts children’s active lifestyles with travel journeys for school children, home to school distance, AST occurrence, as well as the effect of the neighbourhood environment and other influences for three cities within developed economies but with variant urban form and travel characteristics. This study will explore and compare these for children living in Glasgow, Edinburgh and Hong Kong. Despite many differences between the cities, evidence suggests that the effect of urban environments, such as green space within low-income areas and a range of health outcomes, presents similar associations within Hong Kong, Edinburgh and Glasgow (Xu et al. 2017). There is also strong empirical evidence that culture, particularly in Hong Kong middle-classes, predicts behaviours, such as AST and participation in extracurricular activities, as opposed to the built environment (Karsten 2015). North American studies have highlighted that parental support has the strongest independent correlated of AST, indicating that built environment characteristics alone may not be enough to encourage AST (Mah et al. 2017). This study provides an opportunity to understand if similar effects hold within comparable built environment characteristics, or if ‘high rise’ living, differing social structures or parental influence hold greater influence. Systematic reviews have highlighted that inconsistencies among outcome measures may limit the generalisability of findings (Rothman et al. 2018).

### Conceptual framework, study context and research questions

This section provides a conceptual framework to examine whether AST and formal extracurricular activities are associated with children’s active lifestyles.

#### Conceptual framework

**Figure 1:**
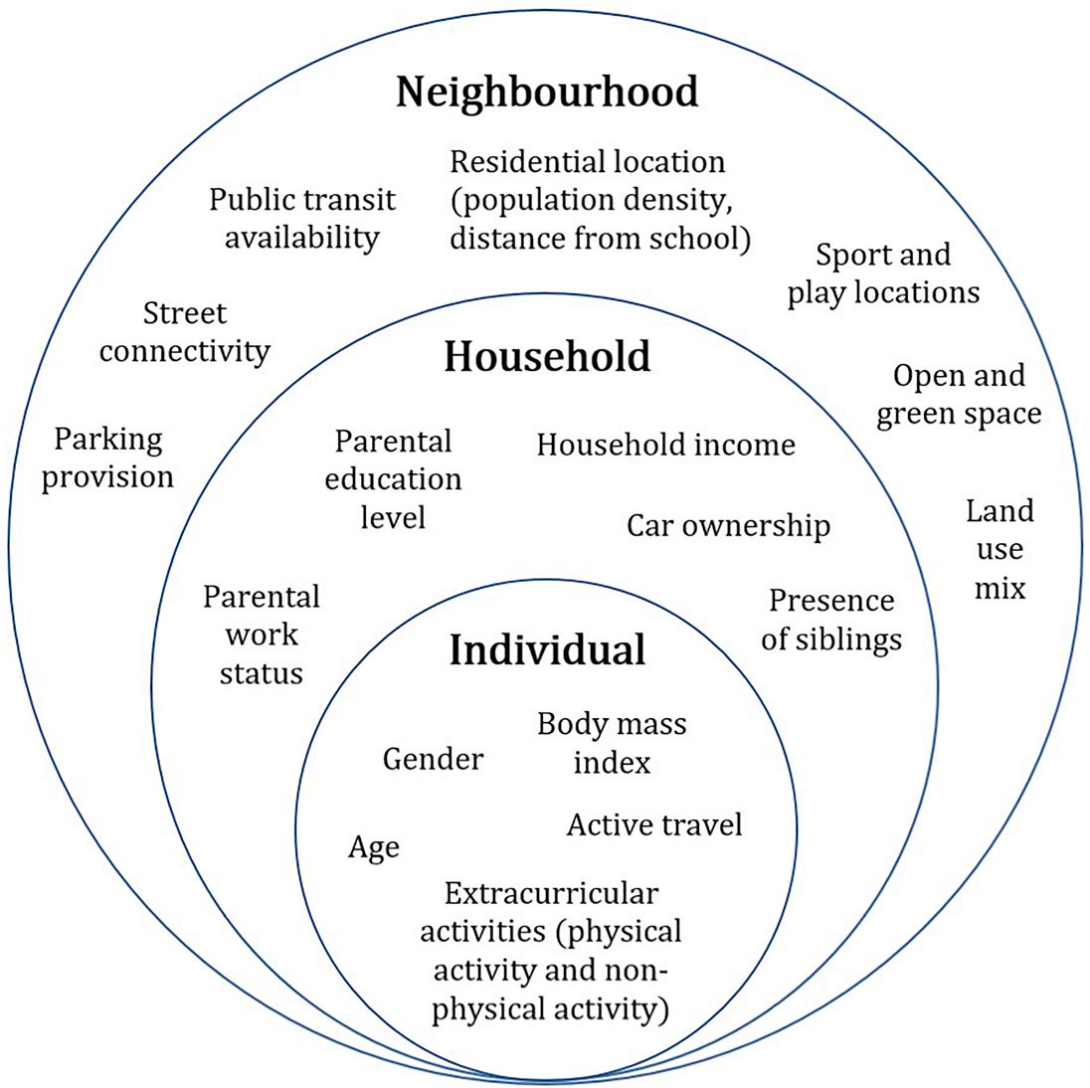
A multi-scale environment-people conceptual framework to understand children’s active lifestyles (adapted from (Loo et al. 2017b, 2017c).

A conceptual framework for the study is shown in Figure 1. This comparative study of three cities examines children’s active lifestyles and their association with the relevant individual, household and neighbourhood-level attributes, with reference to the multi-scale framework suggested in Becky P Y Loo et al. (2017b, 2017c) and in accordance with the discussion in the previous section. The multi-level structure within the conceptual framework consists of three circles that represent the individual scale, the household scale and the neighbourhood scale. Individual-level attributes relate to the participant himself/herself, such as age (in years) and sex (male or female). Their AST and participation in activities (including PA) are also considered under the individual-level, measured in minutes participated, as well as whether they are overweight/obese. Each individual resides within a household, each having household-level attributes such as household car ownership (yes or no), monthly household income (three categories), number of siblings in the household, as well as parental work status (whether one or both parents work). Each household, in turn, is situated within a particular neighbourhood, which will have varying neighbourhood-level attributes such as public transport connections, car parks and recreational facilities (measured by number), street connectivity (measured by intersection density), green spaces (measured by proportion) and land use mix (measured by the presence of more than one land use).

#### Study context

This study considers the active lifestyles of children in three cities: Glasgow (UK), Edinburgh (UK) and Hong Kong (China). The Cities of Glasgow and Edinburgh are the two largest council areas in Scotland (City of Edinburgh population 518,500; Glasgow City 626,410) (National Records of Scotland 2016). Scotland, and in particular Glasgow City, has relatively high mortality rates that are explained by decades of deindustrialisation, deprivation and poverty (Walsh et al. 2017). Both Glasgow (3400 per km^2^) and Edinburgh (1830 per km^2^) are densely populated cities but not on a scale similar to megacities in Europe (e.g. London) or worldwide (e.g. New York City, Hong Kong).

On average, children in Scotland spent 73 min per day in PA (moderate to vigorous), boys exhibit significantly higher levels than girls and there is no socioeconomic patterning (Mccrorie, Mitchell, and Ellaway 2018); these patterns are similar to children living in the rest of the UK and Europe. AST for Scottish children is associated with higher overall moderate to vigorous PA throughout the day compared to children using motorised modes for school travel (Alexander et al. 2005). Car use for travel is high in the UK, chosen due to cultural- or preference-related influences as much as structural factors. Barriers to active travel include those arising from weather and hills (which are prominent in Glasgow and Edinburgh and contribute to their wet climate) (Kirby and Inchley 2009), as well as perceptions of risks of traffic and ‘stranger danger’ (Lorenc et al. 2008). The Scottish Government and Chief Medical Officer (CMO) of Scotland have identified AST as one key way for children to meet their recommended daily physical activity through policies such as *‘A More Active Scotland: Building a Legacy from the Commonwealth Games’* (Scottish Government 2014), which states: ‘Promoting active travel on the school journey can make a positive contribution and complement policy and related initiatives to promote healthy lifestyles and physical activity’.

In contrast, Hong Kong is a highly dense and motorised city with a population of over 7 million and an urban form that is characterised primarily by vertical, rather than horizontal, development (Lau, Giridharan, and Ganesan 2005; Yao and Loo 2016; Leung et al. 2019). Vertical developments in Hong Kong are common in terms of skyscrapers that often also have multiple land uses, such as residential, commercial and transportation, combined within one structure. This verticality can be described as the defining character of the city (Lau, Giridharan, and Ganesan 2005). Given that Hong Kong’s borders are settled by its coastline and northern border with mainland China, lateral, lower-rise urban sprawl-type development is simply not possible, much like the predicament of Manhattan in New York City (Johnson 2020). Hong Kong’s children are typically raised in high-rise apartments within compact neighbourhood environments. Public spaces and facilities available for children’s out-of-home activities are quite limited, perfunctory and lacking in creativity (Karsten 2015; Loo and Lam 2015). The parenting style of Chinese parents is highly protective in nature (Lim and Skinner 2012) and government policy in Hong Kong concurs in recommending that children be supervised by an adult on the road and in the neighbourhood until aged 12 years or older (Transport Department 2018). This Chinese mind-set remains at odds with a number of European countries where high level of independent mobility among children has historically been encouraged and seen as highly desirable, such as in Germany, England and the Nordic countries, but has been declining over the past decades (Shaw et al. 2015).

Some scholars have considered the overarching ‘sport culture’ of a location when investigating PA prevalence (Hertting and Karlefors 2013). Sit, Lindner, and Sherrill (2002) quite bluntly points out that Hong Kong’s culture is *‘not at all a sporty or physically active one’,* where the main emphasis is on academic success coupled with upward mobility in society, and this is further exemplified by Hong Kong’s rather low average moderate to vigorous PA participation (Sallis et al. 2016). Despite this, the busy schedule of sport activities after school and on weekends among Hong Kong children means that they have a considerable level of PA participation coming from their extracurricular activities (K Y.K. Leung et al. 2019). There are also PA opportunities within the local school curriculum as Hong Kong schools are expected to provide two physical education (PE) sessions per week, lasting 35–40 min each, as well as morning and afternoon recesses where children can *‘run around and play at school’* (GovHK 2011).

#### Objectives

Rooted in the conceptual framework, the study objectives were as follows:

(1) Create an active lifestyle variable (ALIFE) measured in terms of total minutes of AST and extra-curricular activities.
(2) Explore how children’s active lifestyles (ALIFE) are associated with different attributes at the individual, household and neighbourhood levels outlined in our conceptual framework, and how these relationships differ across the three cities.
(3) Explore differences in AST and participation in extracurricular activities for children living in Glasgow, Edinburgh and Hong Kong.

## Methods

### Data collection

The data collected for this comparative study of three cities involve two independent cross-sectional surveys and spatial data from two survey databases comprising the three cities of Glasgow, Edinburgh and Hong Kong.

### Surveys

Based on the conceptual framework, comparative variables were identified and obtained from two datasets that contained data of children’s active lifestyles, specifically the child’s school travel characteristics (travel time and main mode), reported extracurricular activities (organised leisure activities outside of school, for example: community group, team or individual sport coaching/lessons and art/performance lessons) (weekly participation duration), residence location (to calculate network distance to school), child’s sex and household socio-economic characteristics (parental work status, income and car ownership).

For the cities of Glasgow and Edinburgh, data are from the *Studying Physical Activity in Children’s Environments across Scotland (SPACES)* study. The *SPACES* study investigated the physical activity of a sub-sample of 10–11-year olds who participated in the eighth sweep of a large-scale cohort study, Growing Up in Scotland (GUS). Data collection took place between May 2015 and May 2016 as part of a follow-up to the *GUS* study and children were asked to complete a detailed travel diary of their daily mode of travel to and from school. More details of the rationale and survey administration procedures are available on the website (http://spaces.sphsu.mrc.ac.uk/), associated publications and the full report (Mccrorie and Ellaway 2017; Olsen et al. 2019). The Scottish data included children from the cities of Glasgow (*N* = 93) and Edinburgh (*N* = 70).

The Hong Kong data are based on a school survey conducted across four primary schools between November 2015 and June 2016. With the help of a team of research assistants, participating children answered a questionnaire and also brought a separate questionnaire home for their parents to fill in. Proportionate stratified sampling, based on private and public schools, has been conducted; and more details about the Hong Kong school survey can be found in K Y.K. Leung and Loo (2017). The Hong Kong children included in this study (*N* = 172) were selected based on the age range (aged 10–11 years) to correspond with the readily available data for the *SPACES* study in Scotland. Both databases also included data about children’s measured height and weight, BMI was calculated to represent the physical profile of each participating child. BMI is considered as a potential associated factor of children’s physical activity and therefore could contribute to their overall active lifestyle (Schwarzfischer et al. 2017).

### Neighbourhood environment

Neighbourhood contextual information was collected mainly from OpenStreetMap (OpenStreetMap 2018) across the three cities for comparison purposes and supplemented by local government sources (Scottish Goverment 2016; Department 2018; Sport Scotland 2018). Although the range of neighbourhood environmental variables available is quite broad, this paper has selectively considered five main categories (as per Wong, Faulkner, and Buliung (2011a)) to include in the analysis: distance to school, neighbourhood density, diversity, design and aesthetics. The data required for calculating distance to school have been discussed in the previous sub-section. Population data were obtained from the 2011 censuses by Tertiary Planning Unit (TPU) in Hong Kong (Census and Statistics Department 2011) and data zone in Scotland (National Records of Scotland 2016), the smallest comparable data collection units in the respective location, as a measure of population density. In terms of diversity, land use data were obtained to prepare a land use mix measure. For neighbourhood design, the availability of public transit, sport and play facilities and car parks were collected, as was number of intersections. Aesthetics-wise, the proportion of open and green space areas (including parks and playgrounds) was calculated as a representation of greenery in the neighbourhood. With reference to the neighbourhood portion of the multi-scale framework of this paper (Figure 1), these chosen neighbourhood environment variables have been adapted and improved from Kevin YK Leung and Loo (2020), which utilised a selection of these variables in their study on AST in Hong Kong.

### Data processing and analysis

This section describes the preparation and processing of the variables for data analysis, as well as the methods of analysis used. For all variables we selected datasets and procedures that could be performed in all three cities using the same contextual data.

### Preparation of neighbourhood environment variables

For data preparation, neighbourhood-level data were organised into the variables outlined above. A detailed description of the built environmental variables and sources are contained within Supplementary Table S1. Spatial coordinates of home and school locations were used to calculate network walking distances between these locations using the *gmapsdistance* package in Rv3.5.1 (the shortest walking distance calculated using the Google Maps distance matrix API using road and path network). For each residence location, 500-metre Euclidean buffers were created in ESRI Arc-Map 10.3, as respective estimations of the immediate neighbourhoods (Loo, Cheng, and Nichols 2017a; Loo and Du Verle 2017), which is a commonly used boundary in studies of the built environment. Buffer analysis was conducted to find the number of *points* and proportion of *polygons* within the buffer areas, and overlaid on the respective Glasgow, Edinburgh and Hong Kong base maps to represent the neighbourhood form surrounding children’s home locations, namely public transit availability, public sport and play locations, car parking facilities, open and green space area and number of road intersections, as a proxy measure for street connectivity (Wong, Faulkner, and Buliung 2011a; Wang and Wen 2017). A land use mix measure was created to include whether the home neighbourhood included an education, government or commercial facility (inconsistent residential building data meant we were unable to create a more sophisticated measure, such as a Diversity Index). Population density was calculated by dividing the 2011 census population by the area for the TPU or data zone within which each child resided.

### Statistical analysis

To study active lifestyles as conceptualised in the framework illustrated in Figure 1, we created an active lifestyle variable (ALIFE). The ALIFE variable measured the total number of minutes of AST and extracurricular activities per week for individual children. The ALIFE variable was calculated by totalling the weekly minutes spent in extracurricular activities (all participants, as captured in the survey) with the daily home to school walking time calculated for children who reported AST for all journeys (multiplied by 5 for a weekly measure).

For the main continuous study outcome, a linear regression model was used. The secondary outcome of interest was participation in AST, as a binary variable (yes/no) for all journeys to and from home to school. As this outcome was binary, logistic regression models were used. To improve the precision of the standard errors of the coefficients of the models, bootstrapping was also applied, at 500 repetitions. Originally, to account for possible similarity between the Scottish cities, clustering on region was considered, but this resulted in over-specification of the models. Multi-level modes were not performed as we did not have sufficient sample size at the country (n:2) or city level (n:3). Evidence has suggested error and bias resulting from multi-level models where groups are less than 50 (Maas and Hox 2005). We, therefore, used fixed effects as we were interested in possible region/city-specific influences. We computed the variance inflation factor (VIF) for each of the final models to test for multi-collinearity of variables, which were all less than 10. In all the main effect-only models, VIF was under 5. In the interaction models where extra inter-variable correlation is likely, the VIF was under 10.

For both the main and secondary outcome models, two sets of models were run, one including region, and the other, city. All independent variables were entered in the model, then simplified by removing non-significant terms (at 0.05 level) individually, highest p-values first. Of the independent variables, only sex was treated as a confounder and always retained in the model. Any variables that were found to be significant at the end of the selection process were checked for interaction with city/region. From these models, interaction plots were produced to explore the effect of these significant variables and how they may moderate AST. All analyses were performed in StataSE 14, and alpha was taken as 5%.

## Results

Overall, the mean number of minutes per week children, participated in AST and extracurricular activities combined within the ALIFE variable, was 355 min. ALIFE varied by city, where children in Glasgow had greater ALIFE than Edinburgh and Hong Kong (Table 1). There was variation in ALIFE by household income, children in the highest income category displaying 114.6 mean minutes greater than those in the lowest income category. AST contribution to total ALIFE (mean by city) varied: 11.1% (Glasgow), 18.6% (Edinburgh) and 13.7% (Hong Kong) (Table 2). Children, whose parents owned a car and were both employed, had a higher mean ALIFE.

The mean AST (all trips to and from school active) for all cities was 37.4%, Hong Kong children reported lower than average AST (34.3%) (Table 1). Glasgow was similar to average AST (39.8%) and children living in Edinburgh displayed the highest AST (47.1%). AST was higher for children who had a lower household income, where parents did not own a car, and if both parents did not work. The characteristic with the greatest change in AST between categories was distance to school; children living closest to school having higher proportion of AST (64.5%) compared to those living over 2 km away (22.9%).

There was little variation in the home to school travel time (minutes) for children across all three cities (Table 3). The network distance was similar for Glasgow and Edinburgh but notably at least two times higher for children living in Hong Kong. Environmental characteristics that varied and were considerably higher for children’s home environment (500 m) in Hong Kong were number of road junctions, public transit stops and population density. The number of sport and play facilities (public) within 500 m of children’s homes were higher for children living in Glasgow and Edinburgh compared to Hong Kong.

Children in Hong Kong had significantly less weekly ALIFE than those in Scotland (Edinburgh/Glasgow combined: coef: −95.13, 95% CI −140.1 to −44.2) and Glasgow (coef: −109.36, 95% CI −181.13 to −37.59) (Table 4). The main effect model showed that household income held a significant influence over a child’s ALIFE, a high-income household having a higher mean weekly ALIFE compared to lower income (coef 115.3, 95% CI 41.68–188.8). There were no difference in ALIFE between boys and girls. Few of the built environment and individual levels showed significant effect on ALIFE, the full model including all variables is presented in Supplementary Table S2.

Testing a model, which included only city, and one that included only region, indicated no differences between cities (0.125) or regions (0.072). Examination of the responses indicated that city was a three-level version of region, which would explain the model over-specification that arose when trying to cluster on city. Therefore, Glasgow and Edinburgh were combined for the final analysis and presented in Table 5. Few of the built environment and individual levels showed significant effect on AST, the full model including all variables is presented in Supplementary Table S3.

Children living in Hong Kong had lower odds of AST compared to Scotland (OR: 0.52, 95% CI 0.29–0.93), for households that owned a car (OR: 0.40, 95% CI 0.21–0.76) and for areas with a greater number of parking facilities around the home (OR: 0.89, 95% CI 0.82–0.97) - this may be an indicator of densely populated areas (Table 3). Distance from home to school was an important predictor of AST, where the likelihood of AST decreased as home to school distance increased. Participation in extracurricular activities was not a predictor for AST in our model.

**Figure 2:**
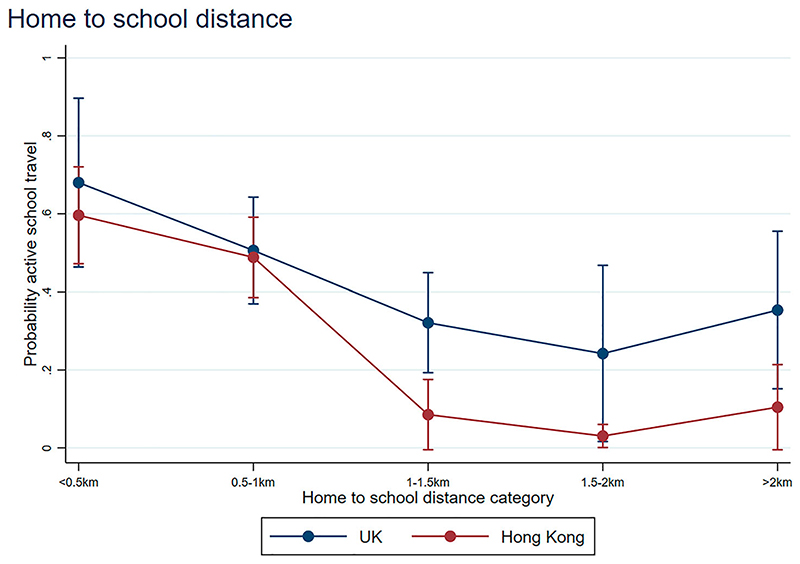
Probability of active travel by home to school distance and region.

**Figure 3:**
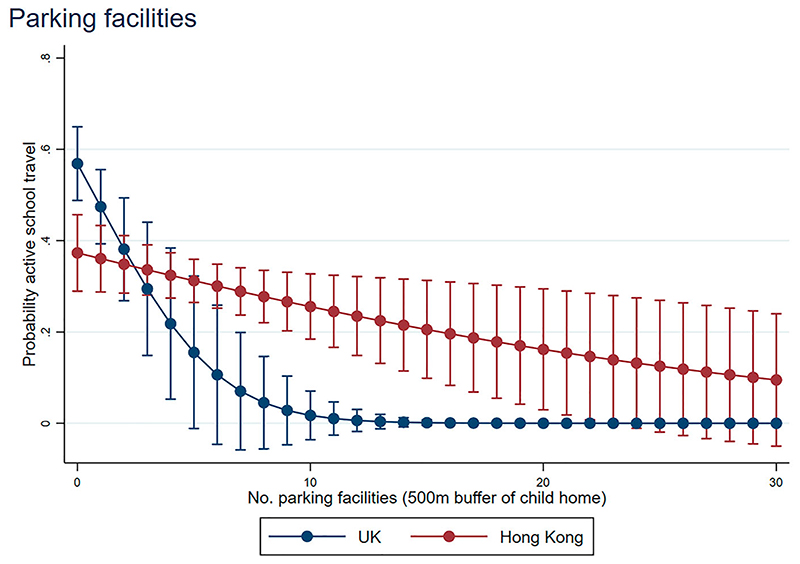
Probability of active travel by number of parking facilities around home.

Figures 2 and 3 display interaction plots of the marginal effects, illustrating the predicted probability of AST by number of parking facilities (within 500 m of child’s home) and by each distance category. In general, the odds of AST were higher where the distance travelled is shorter, as expected (Figure 2). However, it is interesting that (1) increasing number of parking facilities decreases the probability of AST within both countries distance categories; and (2) this is not a constant increasing linear effect in Hong Kong, with reduced gains after a certain point (Figure 3).

## Discussion

### Main findings

This study aimed to create, and created, a children’s active lifestyles (ALIFE) variable for use in international comparison studies that combined weekly number of minutes actively travelling to and from school (AST) and participating in extracurricular activities. Using this novel ALIFE variable, we were able to answer our second research question which explored whether children’s active lifestyles were associated with different individual, household and neighbourhood attributes. We found that children in Glasgow, Edinburgh and Hong Kong had a mean weekly ALIFE of 355 min (5.9 h). ALIFE varied by city, those living in Glasgow and Edinburgh participated in over an hour more ALIFE per-week than those in Hong Kong. We found the most substantial determinant of ALIFE was household income, those from the lowest household income group having almost 2 h less ALIFE per-week than those from the highest income.

Our final study aim was to explore differences in AST and participation in extracurricular activities for children living in Glasgow, Edinburgh and Hong Kong. We found there were city-level differences in the overall proportion of AST between Glasgow, Edinburgh and Hong Kong. Where the data were pooled at a regional level, we found the odds of AST were 48% lower in Hong Kong, once adjusted for individual and household level variables. Participation in extracurricular activities did not influence levels of AST across any of the three cities.

### Comparison with the existing literature

Here, we considered multiple factors that contribute to a child’s total active lifestyle (ALIFE); AST and extracurricular activities, as well the influence of home location and neighbourhood characteristics. Recent studies have explored whether energy used via PA during daily travel may have a direct impact on other daily activities, for example less need or time for other kinds of activity, as well as spending time within neighbourhood green space (Stefansdottir, Næss, and Ihlebæk 2019). A study of adults concluded that the most significant factor for a physically active lifestyle was distance from the central business areas, an association with household income was not found. Here, we found that household income was the most significant influence of ALIFE for children. A previous study of Hong Kong children found that households with more educated parents and with a higher income participated in more extracurricular activities (Lau and Cheng 2016). This pattern was similar in the UK context where disadvantaged children were less likely to take part in organised out-of-school activities, specifically for activities where additional parent/guardian payment was required (music lesson, sport club and extra tuition) (Tanner et al. 2016) – for pre- and post-school clubs, there was similar uptake regardless of income. It is important to note that our study captured only ‘formal’ extracurricular activities and participation in informal outdoor play and sport may differ by household income. We recommend further research captures the specific extracurricular activity.

Similar to other studies, home to school distance had a significant influence on the likelihood of AST in all three cities but not ALIFE, we found the likelihood of AST decreased for children living over 1 km from their school. A study of 10-year-old English school children found the threshold distance that best discriminated walkers from passive commuters was 1.4 km (Chillón et al. 2015). This finding was similar to the distance found here that highlighted a change in the likelihood of AST. Home to school distance has been found to be the most significant environment factor for AST in a large number of studies (Curtis, Babb, and Olaru 2015; Zhang, Yao, and Liu 2017; Rothman et al. 2018), and research has suggested that for primary school-aged children this is often due to parents considering over 20 min beyond their child’s capacity to walk on a school day (Ahern et al. 2017). Further research is required to better understand whether children are actually capable, or even enjoy walking these distances (or longer distances) to school. We tested a number of built environment and individual variables within our model and found few held importance for both ALIFE and AST. It may be helpful to look to the literature to explain this result, such as Timperio et al. (2004) or Handy, Cao, and Mokhtarian (2006), that both found perceptions of good recreational and public transit facilities in the neighbourhood to be associated with more active behaviour. Consequently, it isincreasingly important to consider not only the quantity of neighbourhood facilities but also perceived quality. Further research is required for better understanding how people perceive the convenience of access public transit, recreational facilities and green spaces, in order to more comprehensively consider how active lifestyles are associated with the neighbourhood environment.

Children from households reporting car ownership had a 60% lower likelihood of AST than those without car ownership. Car ownership is socially patterned where individuals from lower socio-economic position are less likely to own a car (Kingham, Pearce, and Zawar-Reza 2007). However, our research suggests that not owning a car can provide an unintended health benefit through increased PA and time spent outdoors being active, potential contribution to protecting and improving health for those whose health status tends to be worse, this result is similar to previous a previous study of Scottish adults’ active travel (Olsen et al. 2017). Australian research has shown that disadvantaged neighbourhoods have more exposure to traffic but greater connectivity and transit access (Rachele et al. 2017). Street connectivity was measured in our study by proxy using road junction density. We found an increased likelihood of AST travel in areas with higher road junction density, corroborating findings in the scientific literature and confirming the importance of fine-grained, pedestrian-friendly urban design (Pojani and Boussauw 2014). In Glasgow 2% of children reported they did not have access to a car, this small number of children (exact number not reported to avoid potential disclosable information) also reported AST for all journeys to and from school. Although this may seem surprisingly high, this may be expected for two reasons, firstly very small numbers in this sub-sample, and secondly, in Glasgow, the average distance to a primary school is small and more likely to be travelled actively (72% of Scottish children aged between 4 and 11 travelled less than 2 km to school (Transport Scotland 2016)).

On a closely related note, we found that where the number of parking facilities increased, the probability of AST rapidly decreased and other research has suggested that parking availability in Asia is associated with more car trips (Yin, Shao, and Wang 2018). The authors of this study also suggested that implementing parking restrictions at journey origins and destinations could encourage more sustainable travel, while our study goes one-step further in suggesting that this could also promote more travel that is active through discouraging car use (Yin, Shao, and Wang 2018).

### Strengths and weaknesses

Our study has a number of strengths. The analysis utilised a comparable sample of school-aged children from two cities in Scotland and Hong Kong, meaning that although the design is cross-sectional, therefore, unable to ascertain causality, they provide similar results in contrasting geographic contexts that further the generalisability of our findings. We were able to create a new active lifestyle variable (ALIFE) for children living in three cities, this variable combined number of minutes travelling to and from school and participating in extracurricular activities. We were unable to measure participation in physically active extracurricular activities only, which is an important dimension of children’s total extracurricular activities (Smith and Haycock 2016) and should be investigated further.

There were a number of similarities within the datasets, meaning a comparable ALIFE and AST outcome, and individual and neighbourhood characteristics were generated. We included a number of directly comparable variables identified in our theoretical framework that were suitable for international comparison and different contexts, which is a key strength of this study. We did not have access to a comparable walkability measure and instead used a measure in road junction density.

In terms of limitations, travel mode was determined via travel diary entries which may not be as accurate as objectively measured mode of transport. Our study may be limited by the inclusion of shortest home-to-school distance rather than actual distance which children travelled. The lack of differences seen between cities could be due to the number of participants in the Scottish cities, which were both around half of Hong Kong’s, meaning a lack of power to detect them, especially as when combined some regional level differences were detected. For the ALIFE variable, we did not know the number of minutes children travelled to and from school actively. However, we were able to create this figure using shortest network distances which estimates the number of minutes an adult would take to walk to and from a destination for children who reported they actively travelled all of their school journeys. By using the shortest network distance and adults’ travel speeds, it is likely the ALIFE variable produced a conservative figure.

## Conclusions

This cross-region multi-city study of school children living in Glasgow, Edinburgh and Hong Kong found that the most substantial contribution to children’s overall active lifestyle was household income, those from the lowest household group having almost 2 h less ALIFE than those from the highest income. A novel ALIFE variable was created that is well suited for use in international comparisons and different contexts. Distance to school was the most significant environmental indicator of AST in both geographical contexts. We found other environmental factors to be important indicators, for example parking facility density, and contribute to the wider base evidence by suggesting parking restrictions at journey origins and destinations could encourage more AST.

## Supplementary Material

Supplementary table 1

Supplementary table 2

Supplementary table 3

## Figures and Tables

**Table 1:** Participant characteristics by city, main children’s active lifestyles (ALIFE) and secondary Active School Travel (AST) study outcome.

Participant characteristics		Glasgow				Edinburgh				Hong Kong			
		*N*	%	ALIFE (mean minutes)	% AST	*N*	%	ALIFE (mean minutes)	% AST	*N*	%	ALIFE (mean minutes)	% AST
Sample size	City	93	20.7	422.7	39.8	70	15.6	401.3	47.1	286	63.7	321.9	34.3
Sex	Male	49	52.7	367.8	28.6	29	41.4	375.2	55.2	159	55.6	349.7	37.7
	Female	44	47.3	483.9	52.3	41	58.6	419.8	41.5	127	44.4	286.5	29.9
Age	10	33	35.5	406.1	27.3	25	35.7	336	36.0	130	45.5	342.8	35.4
	11	60	64.5	431.8	46.7	45	64.3	437.6	53.3	156	54.5	304.0	33.3
Household Income (GBP)	<19,999	10	10.8	458.7	70	-	-	238.6	44.4	32	11.2	229.9	40.6
	20,000–37,999	22	23.7	353.6	31.8	-	-	317.6	56.3	69	24.1	318.2	40.6
	≥38,000	53	57.0	464.6	39.6	-	-	467.0	46.5	167	58.4	347.9	31.1
	Missing	*8*				-				*0*			
Car ownership	Yes	-	-	425.4	38.5	-	-	420.6	44.4	148	51.7	329.2	23.4
	No	-	-	301.2	100.0	-	-	227.9	71.4	138	48.3	313.6	40.6
Overweight/Obese	Yes	17	18.3	444.0	35.3	24	34.3	411.1	58.3	79	27.6	342.0	39.2
	No	75	80.6	416.3	40	45	64.3	392.8	40	201	70.3	316.0	32.8
Number of other children in household	0	-	-	454.2	31.3	-	-	384.3	41.7	12	4.2	375.6	50.0
	1	-	-	425.0	40.4	-	-	417.2	41.0	122	42.7	340.5	28.7
	2	-	-	422.3	42.9	-	-	383.1	58.8	124	43.4	302.5	37.1
	>3	-	-	355.6	44.4	-	-	347.9	100.0	28	9.8	301.3	39.3
Employment status (both parents work)	Yes	-	-	414.2	36.1	-	-	421.4	46.0	195	68.2	341	32.8
	No	-	-	498.8	66.7	-	-	220.6	57.1	88	30.8	280.3	37.5
Distance (home to school, network distance (km))	<0.5 km	-	-	414.8	75.0	-	-	371.2	60.0	-	-	276.9	63.4
	0.5 to 1 km	-	-	370.8	55.2	-	-	338.3	48.2	-	-	346.8	47.4
	1 to 1.5 km	-	-	416.4	29.6	-	-	344.2	35.3	-	-	327.6	7.7
	1.5 to 2 km	-	-	444.5	10.0	-	-	476.9	42.9	-	-	344.9	2.6
	>2 km	-	-	526.3	20.0	-	-	672.9	47.1	-	-	340.6	12.5

Notes: The annual household income categories in the Scottish survey correspond with the categories of <180,000, 180,000 HKD to 455,999 and >456,000 HKD in the Hong Kong survey. No narrower categories were appropriate, the GBP-HKD currency conversion corresponded with the exchange rate at the time of the survey (in late 2015 to early 2016). – numbers below 10 suppressed across all categories to avoid potential disclosable information.

**Table 2:** Contribution of AST to ALIFE, by city.

City/Region	Mean proportion of Active School Travel (AST) within children’s active lifestyles (ALIFE) variable	Standard Deviation	Range
Glasgow	11.1	17.3	0–80.9
Edinburgh	18.6	27.6	0–100
Hong Kong	13.7	25.6	0–100

**Table 3:** City characteristics.

City	School environment			Home environment (500m)				
	Home-school travel time (mins)	Network distance home-school (metres)	Duration of extra-curricular activities (mins)	Road junctions	Public transit stops	Parking facilities	Sport and play facilities (public only)	Population density (persons per sq. km)
Glasgow	19.3	1545.3	354.8	89.74	6.21	0.95	24.4	4346.46
Edinburgh	16.4	1320.1	322.1	88.9	6.44	0.89	36.4	4190.4
Hong Kong	18.5	3565.9	282.9	89.27	17.4	4.78	13.9	31,756.2

Note: Mean values are presented in the table for minutes, metres and number.

**Table 4:** Main effects model – effect of individual and neighbourhood characteristics on children’s active lifestyles (ALIFE) (region and city as fixed effect).

Children’s active lifestyles (ALIFE)		Coefficient	*P*	LL 95% CI	UL 95% CI
(a) Region as fixed effect Region/City	Scotland	Ref			
	Hong Kong	−95.13	0.00	−146.09	−44.17
Sex	Male	Ref			
	Female	−11.09	0.62	−55.18	33.00
Annual household income (thousand GBP)	<20,000	Ref			
	20–40	50.80	0.21	−29.26	130.86
	40+	115.26	0.00	41.68	188.83
(b) City as fixed effect City	Glasgow	Ref			
	Edinburgh	−32.29	0.45	−116.88	52.29
	HK	−109.36	0.00	−181.13	−37.59
Sex	Male	Ref			
	Female	−9.85	0.68	−55.93	36.24
Annual household income (thousand GBP)	<20,000	Ref			
	20–40	50.17	0.21	−28.47	128.80
	40+	114.95	0.00	43.55	186.34

**Table 5:** Effect of individual and neighbourhood characteristics on active school travel (AST) (Region as fixed effect).

Active school travel (AST)		Odds Ratio (OR)	*P*	LL 95% CI	UL 95% CI
Region/City	Scotland	Ref			
	Hong Kong	0.52	0.03	0.29	0.93
Gender	Male	Ref			
	Female	0.97	0.89	0.61	1.54
Car ownership	No	Ref			
	Yes	0.40	0.01	0.21	0.76
Distance from home to school	<0.5 km	Ref			
	0.5 to 1 km	0.54	0.04	0.30	0.98
	1 to 1.5 km	0.11	<0.001	0.06	0.24
	1.5 to 2 km	0.05	<0.001	0.02	0.17
	>2 km	0.14	<0.001	0.05	0.37
No. public parking facilities (500 m buffer of child residence)	0.89	0.01	0.82	0.97
Road junction density (500 m buffer of child residence)	1.01	<0.001	1.01	1.02
