# Supplementary material for: Do neighbourhood characteristics matter in understanding school children’s active lifestyles? A cross-region multi-city comparison of Glasgow, Edinburgh and Hong Kong
Source: Child Geogr. Author manuscript; Available in PMC 2021 Nov 16. (PMC7611993; doi:10.1080/14733285.2020.1828826)
Supplement: Supplementary table 1 [file EMS138302-supplement-Supplementary_table_1.docx]

Supplementary Table 1: Description of built environmental variables included in model.

| **Neighbourhood environment variable** | **Definition** | **Spatial analysis methods** | **Source** |
| --- | --- | --- | --- |
| Public transit availability | Locations of bus, train, tram and metro stops. | Number of features within 500-metre euclidean buffer of child’s home address. | Source: “Tag: type=public_transport “ OpenStreetMap, 2019. |
| Parking availability | Cars & motor vehicle parking | Number of features within 500-metre Euclidean buffer of child’s home address. | Source: “Tag:amenity = parking” OpenStreetMap, 2019. |
| Open and green space areas | Public park, playing field and playgrounds. | Proportion within 500-metre Euclidean buffer of child’s home address. | Source: “Tag:leisure=playground” & “Tag:leisure=park” OpenStreetMap, 2019. |
| Sport and play facilities | Public sport and play locations | Number of features within 500-metre Euclidean buffer of child’s home address. | Source: “Tag:leisure=sports centre” OpenStreetMap, 2019. |
| Road junction density | Transportation nodes marking road intersections. | Number of features within 500-metre Euclidean buffer of child’s home address. | Source: “Tag: highway=junction” OpenStreetMap, 2019. |
| Land use mix | Education: 'college' OR 'kindergarten' OR 'school' OR 'university'  Government: 'public' OR 'public_building' OR 'hospital' OR 'community_centre'  Commercial: 'commercial' OR 'retail' OR 'shop' OR 'restaurant' OR 'fast_food' | Binary outcome to indicate if 500-metre Euclidean buffer of child’s home address including an education, government and commercial facility (note: inconsistent residential building data meant we were unable to create a more sophisticated measure, such as a Diversity Index) | Source: OpenStreetMap, 2019. |
| Population density | Persons per sq km of child’s residential datazone (scotland) or TPU (HK) | Dividing the 2011 census population by the area for the TPU or data zone within which each child resided. | Source: Population data were obtained from the 2011 censuses by Tertiary Planning Unit (TPU) in Hong Kong (Census and Statistics Department 2017) and datazone in Scotland (National Records of Scotland 2016) |
