# Supplementary material for: Do neighbourhood characteristics matter in understanding school children’s active lifestyles? A cross-region multi-city comparison of Glasgow, Edinburgh and Hong Kong
Source: Child Geogr. Author manuscript; Available in PMC 2021 Nov 16. (PMC7611993; doi:10.1080/14733285.2020.1828826)
Supplement: Supplementary table 2 [file EMS138302-supplement-Supplementary_table_2.docx]

Supplementary Table 2: Main effects model (full) – effect of individual and neighbourhood characteristics on active lifestyle (ALIFE) (region and city as fixed effect)

| **ALIFE** | | **Coefficient** | **P** | **LL 95% CI** | **UL 95% CI** |
| --- | --- | --- | --- | --- | --- |
| **(a) Region as fixed effect** | | |  |  |  |
| Region/City | |  |  |  |  |
|  | Scotland | Ref | | | |
|  | HK | -82.64 | 0.01 | -145.93 | -19.36 |
| Age |  |  |  |  |  |
|  | 10 | Ref | | | |
|  | 11 | -24.19 | 0.34 | -74.28 | 25.91 |
| Sex |  |  |  |  |  |
|  | Male | Ref | | | |
|  | Female | -13.68 | 0.59 | -62.72 | 35.37 |
| Education attainment of responding parent | | | | |  |
|  | Secondary or below | Ref | | | |
|  | Post-secondary | -1.11 | 0.97 | -61.32 | 59.10 |
| Both parents work | |  |  |  |  |
|  | No | Ref | | | |
|  | Yes | 18.99 | 0.58 | -48.69 | 86.67 |
| Number of children in household | | | |  |  |
|  | 0 | Ref | | | |
|  | 1 | 0.21 | 1.00 | -91.52 | 91.95 |
|  | 2 | -47.96 | 0.34 | -145.91 | 49.98 |
|  | >=3 | -39.59 | 0.47 | -146.18 | 66.99 |
| Household income | |  |  |  |  |
|  | low income (<20k) | Ref |  |  |  |
|  | mid income (20k to 40k) | 46.77 | 0.28 | -38.64 | 132.18 |
|  | High income (40k +) | 102.88 | 0.01 | 24.16 | 181.60 |
| Car ownership | |  |  |  |  |
|  | No | Ref | | | |
|  | Yes | -28.38 | 0.45 | -102.67 | 45.91 |
| Distance from home to school | | |  |  |  |
|  | <0.5km | Ref | | | |
|  | 0.5km to 1km | 64.34 | 0.02 | 9.25 | 119.43 |
|  | 1km to 1.5km | 50.41 | 0.20 | -26.78 | 127.61 |
|  | 1.5km to 2km | 93.70 | 0.02 | 14.61 | 172.78 |
|  | >2km | 174.37 | 0.01 | 53.84 | 294.90 |
| Population density | | 0.00 | 0.89 | 0.00 | 0.00 |
| Employment density | | 0.00 | 0.33 | 0.00 | 0.00 |
| Land use mix | | -33.11 | 0.42 | -114.21 | 47.98 |
| No. public transport stops | | 2.31 | 0.30 | -2.04 | 6.65 |
| No. public parking facilities (500m buffer of child res) | | -1.71 | 0.75 | -12.08 | 8.65 |
| Open and green space areas | | -5.31 | 0.25 | -14.39 | 3.77 |
| Sport and play facilities | | 0.80 | 0.09 | -0.13 | 1.74 |
| Road junction density | | -0.21 | 0.64 | -1.12 | 0.69 |
| **(b) City as fixed effect** | | |  |  |  |
| City |  |  |  |  |  |
|  | Glasgow | Ref | | | |
|  | Edinburgh | -37.98 | 0.39 | -124.29 | 48.33 |
|  | HK | -99.30 | 0.01 | -173.34 | -25.25 |
| Age |  |  |  |  |  |
|  | 10 | Ref | | | |
|  | 11 | -24.75 | 0.32 | -73.14 | 23.64 |
| Sex |  |  |  |  |  |
|  | Male | Ref | | | |
|  | Female | -12.15 | 0.63 | -62.05 | 37.75 |
| Education attainment of responding parent | | | | |  |
|  | Secondary or below | Ref | | | |
|  | Post-secondary | -1.80 | 0.96 | -68.56 | 64.96 |
| Both parents work | |  |  |  |  |
|  | No | Ref | | | |
|  | Yes | 18.63 | 0.59 | -49.21 | 86.48 |
| Number of children in household | | | |  |  |
|  | 0 | Ref | | | |
|  | 1 | 0.59 | 0.99 | -88.04 | 89.22 |
|  | 2 | -49.03 | 0.31 | -142.90 | 44.83 |
|  | >=3 | -41.87 | 0.42 | -142.97 | 59.22 |
| Household income | |  |  |  |  |
|  | low income (<20k) | 45.08 | 0.30 | -39.48 | 129.64 |
|  | mid income (20k to 40k) | 102.75 | 0.01 | 30.05 | 175.45 |
|  | High income (40k +) | |  |  |  |
| Car ownership | |  |  |  |  |
|  | No | Ref | | | |
|  | Yes | -30.02 | 0.42 | -103.37 | 43.32 |
| Distance from home to school | | |  |  |  |
|  | <0.5km | Ref | | | |
|  | 0.5km to 1km | 64.71 | 0.02 | 10.20 | 119.23 |
|  | 1km to 1.5km | 49.42 | 0.20 | -25.86 | 124.70 |
|  | 1.5km to 2km | 93.45 | 0.03 | 11.39 | 175.51 |
|  | >2km | 173.54 | 0.01 | 50.80 | 296.29 |
| Population density | | 0.00 | 0.91 | 0.00 | 0.00 |
| Employment density | | 0.00 | 0.32 | 0.00 | 0.00 |
| Land use mix | | -25.35 | 0.55 | -107.82 | 57.12 |
| No. public transport stops | | 2.35 | 0.27 | -1.82 | 6.52 |
| No. public parking facilities (500m buffer of child res) | | -1.70 | 0.74 | -11.83 | 8.44 |
| Open and green space areas | | -5.47 | 0.24 | -14.61 | 3.66 |
| Sport and play facilities | | 0.84 | 0.06 | -0.02 | 1.70 |
| Road junction density | | -0.23 | 0.62 | -1.16 | 0.70 |
