# Supplementary material for: Do neighbourhood characteristics matter in understanding school children’s active lifestyles? A cross-region multi-city comparison of Glasgow, Edinburgh and Hong Kong
Source: Child Geogr. Author manuscript; Available in PMC 2021 Nov 16. (PMC7611993; doi:10.1080/14733285.2020.1828826)
Supplement: Supplementary table 3 [file EMS138302-supplement-Supplementary_table_3.docx]

Supplementary Table 3: Effect of individual and neighbourhood characteristics (full model) on active school travel (Region as fixed effect)

| **Active school travel** | | **OR** | **P** | **LL 95% CI** | **UL 95% CI** |
| --- | --- | --- | --- | --- | --- |
| Region/City | |  |  |  |  |
|  | Scotland | Ref | | | |
|  | HK | 0.49 | 0.04 | 0.24 | 0.97 |
| Age |  |  |  |  |  |
|  | 10 | Ref | | | |
|  | 11 | 1.47 | 0.19 | 0.83 | 2.61 |
| Sex |  |  |  |  |  |
|  | Male | Ref | | | |
|  | Female | 1.07 | 0.80 | 0.62 | 1.86 |
| Duration of all extracurricular activities per week | | | | |  |
|  | Minutes | 1.00 | 0.31 | 1.00 | 1.00 |
| Both parents work | |  |  |  |  |
|  | No | Ref | | | |
|  | Yes | 0.97 | 0.95 | 0.45 | 2.10 |
| Education attainment of responding parent | | | | |  |
|  | Secondary or below | Ref | | | |
|  | Post-secondary | 0.68 | 0.33 | 0.31 | 1.48 |
| Number of children in household | | | |  |  |
|  | 0 | Ref | | | |
|  | 1 | 1.35 | 0.52 | 0.54 | 3.38 |
|  | 2 | 1.45 | 0.46 | 0.54 | 3.88 |
|  | >=3 | 1.19 | 0.77 | 0.37 | 3.87 |
| Household income | |  |  |  |  |
|  | low income (<20k) | Ref |  |  |  |
|  | mid income (20k to 40k) | 1.13 | 0.80 | 0.43 | 2.99 |
|  | High income (40k +) | 0.96 | 0.93 | 0.35 | 2.60 |
| Car ownership | |  |  |  |  |
|  | No | Ref | | | |
|  | Yes | 0.42 | 0.06 | 0.18 | 1.02 |
| Distance from home to school | | |  |  |  |
|  | <0.5km | Ref | | | |
|  | 0.5km to 1km | 0.54 | 0.08 | 0.26 | 1.08 |
|  | 1km to 1.5km | 0.09 | 0.00 | 0.04 | 0.22 |
|  | 1.5km to 2km | 0.05 | 0.00 | 0.02 | 0.18 |
|  | >2km | 0.14 | 0.00 | 0.05 | 0.36 |
| Population density | | 1.00 | 0.69 | 1.00 | 1.00 |
| Employment density | | 1.00 | 0.88 | 1.00 | 1.00 |
| Land use mix | | 0.89 | 0.82 | 0.34 | 2.36 |
| No. public transport stops | | 1.04 | 0.14 | 0.99 | 1.10 |
| No. public parking facilities (500m buffer of child res) | | 0.82 | 0.03 | 0.69 | 0.98 |
| Open and green space areas | | 0.90 | 0.10 | 0.79 | 1.02 |
| Sport and play facilities | | 1.00 | 0.97 | 0.99 | 1.01 |
| Road junction density | | .01 | 0.09 | 1.00 | 1.02 |
